# Comparison of Copper-Tolerance Genes between Different Groups of *Acidovorax citrulli*

**DOI:** 10.3390/microorganisms12040682

**Published:** 2024-03-28

**Authors:** Min Zhang, Mei Zhao, Pei Qiao, Dehua Liu, Qingrong Bai, Wei Guan, Yuwen Yang, Tingchang Zhao

**Affiliations:** 1 College of Plant Protection, Jilin Agricultural University, Changchun 130118, China; minzhang1998@foxmail.com (M.Z.); liudehua199804@foxmail.com (D.L.); bbbqqqrrr@163.com (Q.B.); 2 State Key Laboratory for Biology of Plant Diseases and Insect Pests, Institute of Plant Protection, Chinese Academy of Agricultural Sciences, Beijing 100193, China; qp382391128@outlook.com (P.Q.); guanwei@caas.cn (W.G.); 3 Department of Plant Pathology, College of Plant Protection, China Agricultural University, Beijing 100193, China; zhaomeippath@163.com; 4 National Key Laboratory of Green Pesticide, Guizhou University, Guiyang 550025, China; 5 National Nanfan Research Institute (Sanya), Chinese Academy of Agricultural Sciences, Sanya 572024, China

**Keywords:** *Acidovorax citrulli*, bacterial fruit blotch, copper tolerance, *copA*

## Abstract

*Acidovorax citrulli* populations exhibit genetic and phenotypic variations, particularly in terms of copper tolerance. Group I strains of *A. citrulli* generally exhibit higher copper tolerance compared to group II strains. This study aims to identify genes involved in copper tolerance to better understand the differences in copper tolerance between group I and group II strains. Representative strains pslb65 (group I) and pslbtw14 (group II) were selected for comparison. Deletion mutants of putative copper-tolerance genes and their corresponding complementary strains were constructed. The copper tolerance of each strain was evaluated using the minimum inhibitory concentration method. The results showed that the *copA*, *copZ*, *cueR*, and *cueO* genes played major roles in copper tolerance in *A. citrulli*, while *cusC*-like, *cusA*-like, and *cusB*-like genes had minor effects. The different expression levels of copper-tolerance-related genes in pslb65 and pslbtw14 under copper stress indicated that they had different mechanisms for coping with copper stress. Overall, this study provides insights into the mechanisms of copper tolerance in *A. citrulli* and highlights the importance of specific genes in copper tolerance.

## 1. Introduction

Bacterial fruit blotch (BFB) is a seed-borne bacterial disease caused by *Acidovorax citrulli*, which poses a serious threat to cucurbit crops [1,2,3]. Under hot and humid conditions, BFB spreads rapidly, leading to fruit rot in later stages of the disease and causing serious losses to the watermelon and melon industries [4,5]. *Acidovorax citrulli* populations can be divided into two genetically distinct groups, group I and II [6,7]. Current methods for controlling BFB in the field rely heavily on copper-containing bactericides [8,9]. However, only 2% of group I strains of *A. citrulli* are sensitive to copper sulfate at a concentration of 500 µg/mL (3.13 mM), while all tested group II strains are sensitive [10]. The presence of copper-tolerant strains in *A. citrulli* has made prevention and control more challenging, resulting in an increase in group I strains [10]. Therefore, it is crucial to study the copper-tolerance mechanism of *A. citrulli* and understand the disparity in copper tolerance between group I and II strains. This research can provide valuable insights for BFB prevention and treatment.

Copper is essential for bacterial metabolism, but excessive amounts can be harmful [11]. Bacteria must tightly regulate copper homeostasis in the intracellular environment to maintain metabolism and vitality [12]. *Escherichia coli* has multiple mechanisms for copper tolerance. In *E. coli*, copper ions are bound by the copper-chaperone protein CopZ upon entering the cytoplasm, which directs them to transcription regulators and the copper-exporting P-type ATPase CopA. CopA actively pumps excess copper ions out of the cytoplasm and exports them to the Cus system. The periplasmic copper chaperone CusF assists in removing copper ions from the cell [13]. In addition, copper ions in the periplasm can bind to multicopper oxidase CueO, which converts Cu^+^ to the less toxic Cu^2+^ [13], thus protecting periplasmic enzymes from copper-induced damage. Both the copper efflux P-type ATPase encoded by the *copA* gene and multicopper oxidase *cueO* in *E. coli* are regulated by CueR [13].

Several copper homeostasis genes have been identified in *A. citrulli*, including *copA* (*Aave_0034*), *cueO* (*Aave_1810*), *copZ* (*Aave_0033*), and *cusA* (*Aave_0038*) in Aac5 (group II) [10,14,15]; *copA* (*Aave_0034*) in pslb3 (group I) [10]; *cusA*-like (*Aave_0388*), *cusB*-like (*Aave_0389*), *cusC*-like (*Aave_0387*), *zneB* (*Aave_0039*), *cueR* (*Aave_0032*), *copZ* (*Aave_0033*), *cusB* (*Aave_4663*), *tolC* (*Aave_1811*), and *gntR* (*Aave_2798*) in FC440 (group I) [16,17,18,19]. Previous studies using the plate streaking method have found that *A. citrulli* strain pslb3 (group I) and Aac5 (group II) did not grow at Cu^2+^ concentrations of 4.06 mM and 2.81 mM, respectively [10]. Deletion of the *copA* (*Aave_0034*) gene in both strains resulted in an inability to grow at a Cu^2+^ concentration of 0.94 mM [10]. Li et al. (2014) also used the plate streaking method and found that deletion of the *cueO* (*Aave_1810*) gene in Aac5 (group II) resulted in visibly less colony growth compared to the wildtype strain on plates with a Cu^2+^ concentration above 1.88 mM [14]. Through a growth capacity assay, Liu et al. found that deletion of the *copZ* (*Aave_0033*) and *cusA* (*Aave_0038*) genes in Aac5 (group II) led to a decreased tolerance to copper stress at Cu^2+^ concentrations of 1.25 mM and 2.5 mM, respectively [15]. The copper sensitivity of the wildtype strain FC440 (group I) and the mutant strain was determined by the spot-plating method. Wildtype strain FC440 did not grow at a Cu^2+^ concentration of 7.5 mM, while mutant strain Δ*cusB* (*Aave_4663*) did not grow at a Cu^2+^ concentration of 1.25 mM [17]. Mutant strains Δ*tolC* (*Aave_1811*) and Δ*gntR* (*Aave_2798*) did not grow at a Cu^2+^ concentration of 3.75 mM [18], and mutant strain Δ*copZ* (*Aave_0033*) lost its ability to grow at a Cu^2+^ concentration of 4 mM [19]. The mutant strain Δ*cueR* (*Aave_0032*) has a diminished ability to grow at 3.3 mM [19]. Furthermore, Δ*cusA* (*Aave_0388*), Δ*cusB* (*Aave_0389*), Δ*cusC* (*Aave_0387*), and Δ*zneB* (*Aave_0039*) had diminished abilities to grow at 3.75 mM [16].

However, due to the differences in strains and methods for assessing growth on copper-amended media used in previous studies, direct comparisons cannot be made regarding the effects of these genes on copper tolerance in *A. citrulli* strains. Therefore, this study aims to address this gap by generating mutant and complementary strains based on known copper-tolerance genes from representative group I and group II strains. The roles of these genes in copper tolerance were assessed through measuring the minimum inhibitory concentration (MIC) of copper on solid medium, and the expression levels of copper-tolerance-related genes in the representative strains were compared. This study will shed light on the disparity in copper sensitivity among *A. citrulli* strains, and contribute to our understanding of the mechanisms underlying copper tolerance. Ultimately, the findings of this research will aid in the development of disease prevention and control strategies for BFB.

## 2. Materials and Methods

### 2.1. Strains, Plasmids, and Antibiotics

The strains and plasmids used in this study are listed in Table S1. The media and antibiotic concentrations used in the experiments were prepared as follows. KMB [19] solid medium was composed of the following ingredients per liter: Tryptone 20 g, K_2_HPO_4_ 1.5 g, MgSO_4_ 1.5 g, agar 15.0 g, and 1 L H_2_O. The antibiotic concentrations used were ampicillin (Amp) at 100 µg/mL, kanamycin (Kan) at 50 µg/mL, and chloramphenicol (Cm) at 25 µg/mL.

### 2.2. Selection of Copper-Tolerant Representative Strains

Based on the copper sensitivity test conducted by Zhao [10], two strains (pslb9 and pslb65) from group I and four strains (pslbtw14, pslbtw32, pslbtw38, and Aac5) from group II were selected for further screening of representative strains. Copper sulfate (CuSO_4_·5H_2_O) concentrations ranging from 0 to 7.2 mM were used for the screening process. The strains were cultured in KMB liquid medium at 28 °C and 220 r/min for 12 h. Bacterial suspensions with an OD_600_ of 0.3 (equivalent to 3 × 10^8^ CFU/mL) were mixed with KMB liquid medium containing copper in a ratio of 1:100. The mixture was then added to 100-well polystyrene plates, and incubated at 28 °C with shaking in a Bioscreen C for monitoring growth (Bioscreen C° PRO, Helsinki, Finland). The OD_600_ values were measured every 2 h. The experiment was conducted three times.

### 2.3. Construction and Verification of Deletion Mutants and Complementary Strains for Putative Copper-Tolerance Genes

Primers were designed using Primer Premier 5.0 (Canada). The DNA from pslb65 and pslbtw14 served as templates. The upstream and downstream fragments of the *Aave_0032* (*cueR*) gene were amplified using primer pairs 0032-1F/1R and 0032-2F/2R, respectively. The resulting fragments were then fused using Overlapping PCR [20,21]. The fused fragment was ligated with pK18*mobsacB* [22,23], which had been digested with *Eco*RI and *Hin*dⅢ. The ligated construct was then transformed into DH5α. The suicide-recombinant vectors were verified using primers M13F/M13R and sequencing. The mutants were constructed using the homologous recombination method with three parental strains and screened with sucrose [24,25,26]. The mutants were validated using *A. citrulli-specific* primers WFB1/WFB2 [27] and target-gene-specific primers 0032-F/R. The validated mutants were sub-cultured and stored. The mutants for the other 11 genes were obtained using the same procedure.

To complement the mutants with impaired copper sensitivity, pslb65 was used as a template. Primer pair H0032F/H0032R was used to amplify *Aave_0032*. The amplified fragment was then ligated with pBBR1MCS-2, which had been digested with *Bam*HI/*Hin*dIII. After confirming the correct complementary vector, it was introduced into the mutants Δ65-0032 and Δ14-0032 through three-parental hybridization. Verification was performed using target-gene-specific primers and Kan primers. Similar methods were used to obtain complementary strains for other genes. The primers used are listed in Table S2.

### 2.4. Determination of Copper MIC

The tested strains were cultured in KMB liquid medium at 28 °C and 220 r/min for 12 h. The bacterial suspensions were adjusted to an OD_600_ of 0.3 and diluted to a concentration of 3 × 10^4^ CFU/mL. Then, 10 µL of the bacterial suspension was spotted on KMB solid medium containing various concentrations of CuSO_4_·5H_2_O (0, 0.1, 0.2, 0.3, 0.4, 0.8, 1.2, 1.6, 2.0, 2.4, 2.8, 3.2, 3.6, 4.0, 4.4, 4.8, 5.2, 5.6, 6.0, and 6.4 mM) [28]. The plates were incubated at 28 °C for 6 days, and the MIC value was determined as the lowest concentration of copper sulfate that showed no bacterial growth. Each strain was tested in triplicate, and the experiment was conducted three times.

### 2.5. Analysis of Gene Expression Related to Copper Tolerance

The single colony of pslbtw14 and pslb65 was cultured in KMB liquid medium with different concentrations of Cu^2+^ at 28 °C and 220 r/min for 12 h. For pslbtw14, the Cu^2+^ concentrations were 0.8 mM and 2.0 mM, while for pslb65, the concentrations were 2 mM and 4 mM, with a control group receiving no copper treatment. The internal reference gene used was the *rpoB* gene. Genes expression levels related to copper metabolism were determined using the relative quantitative method (2^−ΔΔCT^) [29], with the expression levels of each gene in wildtype strains pslbtw14 and pslb65 without copper treatment set as 1 for comparison.

Δ65-0032 and Δ14-0032 were cultured in KMB liquid medium containing 1.6 mM Cu^2+^ for 12 h, with pslb65 and pslbtw14 treated with 1.6 mM Cu^2+^ serving as controls, respectively. The internal reference gene used was the *rpoB* gene. The gene expression levels in pslb65 and pslbtw14 treated with 1.6 mM copper were set as 1 for comparison, and the expression levels of related genes in Δ65-0032 and Δ14-0032 were calculated accordingly.

Total RNA was extracted from the cultures using a bacterial total RNA extraction kit (Yeasen, Shanghai, China). RNA reverse transcription was performed using the FastKing gDNA Dispelling RT Supermix kit (TIANGEN). The primer sequences for quantitative real-time PCR (qRT-PCR) are listed in Table 1.

### 2.6. Data Analysis

The experimental data were recorded and analyzed using Excel 2016 (Microsoft, Redmond, WA, USA). Statistical analysis and graphs were plotted using GraphPad Prism 9 (GraphPad, San Diego, CA, USA). Two-way analysis of variance (ANOVA) in GraphPad Prism 9 was used to determine the significance of gene expression levels related to copper metabolism following different copper treatments (with a confidence interval of 95%). Dunnett’s multiple comparisons test was applied for further analysis.

## 3. Results

### 3.1. Identification of Copper-Tolerant A. citrulli Strains

**Figure 1:**
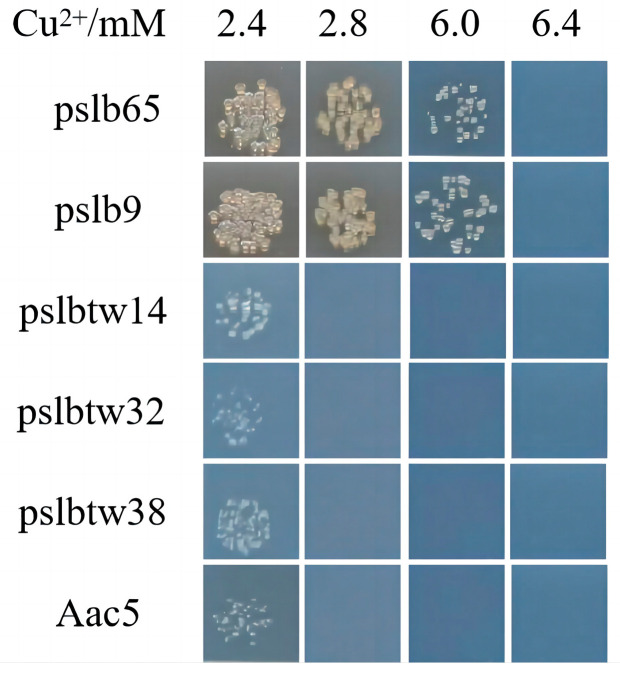
Colony growth of *Acidovorax citrulli* strains pslb65, pslb9, pslbtw14, pslbtw38, pslbtw32, and Aac5 on KMB solid medium with varying copper concentrations after 6 days of incubation. Strains pslb65 and pslb9 belong to group I, while pslbtw14, pslbtw38, pslbtw32, and Aac5 belong to group II. The strains were cultured in KMB liquid medium at 28 °C and 220 r/min for 12 h. The bacterial suspensions were diluted to 3 × 10^4^ CFU/mL and 10 µL of the bacterial suspension was spotted on KMB solid medium containing varying concentrations of CuSO_4_·5H_2_O. The picture shows the colony growth after 6 days of incubation.

**Figure 2:**
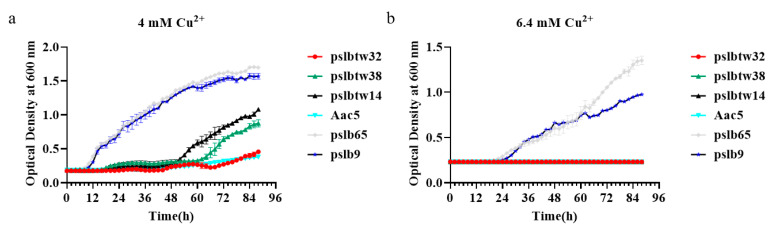
Growth comparisons of *A. citrulli* wildtype strains under different Cu^2+^ concentrations. The growth curves of the tested strains were measured at 4 mM Cu^2+^ (**a**) and 6.4 mM Cu^2+^ (**b**). Strains pslb65 and pslb9 belong to group I, while pslbtw14, pslbtw38, pslbtw32, and Aac5 belong to group II. Bacterial suspensions with an OD_600_ of 0.3 (equivalent to 3 × 10^8^ CFU/mL) were mixed with KMB liquid medium containing copper in a ratio of 1:100. The mixture was incubated at 28 °C with shaking in a Bioscreen C. The OD_600_ values were measured every 2 h.

The copper tolerance of six selected *A. citrulli* wildtype strains was determined by measuring their copper MIC values (Figure 1). Group I strains had copper MIC values of 6.4 mM, while group II strains had copper MIC values of 2.8 mM. This indicated that group I strains were less sensitive to copper than group II strains. Additionally, the growth curves of the selected strains were measured in vitro at different copper concentrations. Clear differences were observed between group I and II strains at copper concentrations of 6.4 mM and 4 mM (Figure 2). At 6.4 mM, the growth rates of the two group I strains were similar within the 0~60 h timeframe. However, after 60 h, the growth of strain pslb65 surpassed that of strain pslb9 (Figure 2b). At a copper concentration of 4 mM, strain pslbtw14 exhibited higher growth than the other group II strains after 48 h (Figure 2a). Therefore, pslb65 and pslbtw14 were selected as representative strains for subsequent experiments.

### 3.2. Verification of Deletion Mutants and Complementary Strains for Putative Copper-Tolerance Genes

Deletion mutants for copper-tolerance genes were constructed using *A. citrulli* group I strain pslb65 and group II strain pslbtw14 as templates. The correctness of the deletion mutants was confirmed using target-gene-specific primers and *A. citrulli*-species-specific primers (Figures S1 and S2). Complementary strains were constructed using DNA from pslb65 as the template and verified using target-gene-specific primers and Kan primers (Figures S3 and S4).

### 3.3. Copper-Tolerance Phenotypes of Representative Strains and Mutants

**Figure 3:**
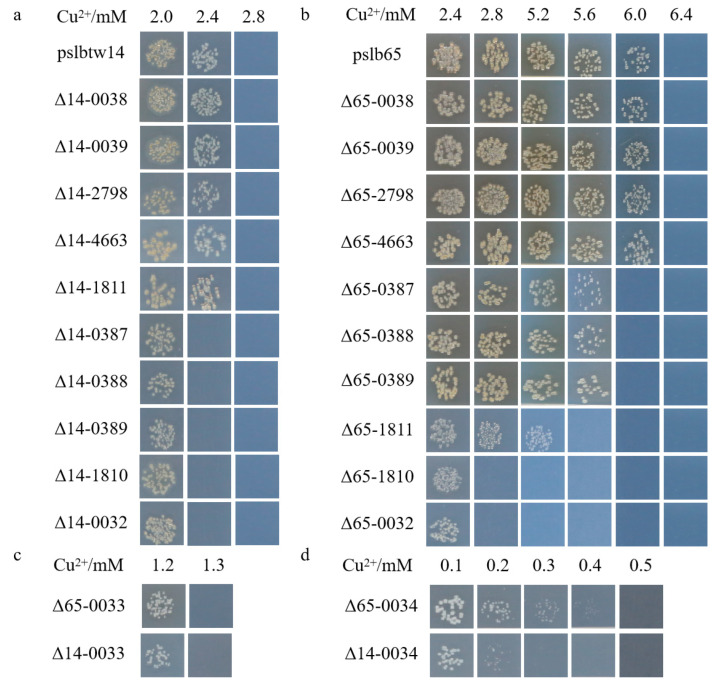
Colony growth of the tested strains on KMB solid medium with varying concentrations of copper. (**a**) Colony growth of representative *Acidovorax citrulli* group II wildtype and mutant strains. (**b**) Colony growth of representative *A. citrulli* group I wildtype and mutant strains. (**c**,**d**) Colony growth of mutant strains at lower copper concentrations. The strains were cultured in KMB liquid medium at 28 °C and 220 r/min for 12 h. The bacterial suspensions were diluted to 3 × 10^4^ CFU/mL. Subsequently, 10 µL of the bacterial suspension was spotted on KMB solid medium amended with varying concentrations of CuSO_4_·5H_2_O. The picture shows the colony growth after 6 days of incubation. Δ65-0032 represents the *Aave_0032*-gene-deletion mutant strain from pslb65. Δ14-0032 represents the *Aave_0032*-gene-deletion mutant strain from pslbtw14. Similar notations apply to the other strains.

**Figure 4:**
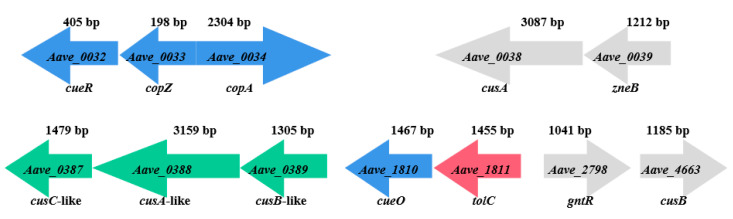
A schematic representation of the organization of copper-tolerance genes in the *Acidovorax citrulli* genome AAC00-1 (Genbank accession NC_008752.1). Genes with a major impact on copper tolerance are shown in blue; genes with a minor influence are shown in green. Genes that may not be directly associated with copper tolerance are shown in gray. The *tolC* (*Aave_1811*) gene shown in red exhibited a reduction in copper tolerance only when deleted in pslb65. The Cue system in *E. coli* is composed of CopA, CueO, and CueR, with *copA* and *cueO* being regulated by CueR. The Cus system is composed of CusA, CusB, CusC, and others. CopA primarily exports copper ions from the cytoplasm, while Cus system exports copper ions from the periplasm to maintain copper balance in the cell. CueO may oxidize toxic Cu^+^ ions in the periplasm to the less toxic Cu^2+^ ions. The mutant strain MIC results in *A. citrulli* suggest a similar copper-tolerance mechanism to that of *E. coli*.

The mutants of representative strains pslb65 (group I) and pslbtw14 (group II) with the same gene deletion exhibited different levels of sensitivity to copper. Strain pslb65 had a copper MIC value of 6.4 mM, and the deletion of certain genes associated with copper tolerance in pslb65 decreased its tolerance to Cu^2+^ (Figure 3). For instance, Δ65-0034 (*Aave_0034*-gene-deletion mutant derived from pslb65, similar below) failed to grow at a copper concentration of 0.5 mM, Δ65-0033 failed to grow at 1.3 mM, and Δ65-0032 and Δ65-1810 failed to grow at 2.8 mM (Figure 3b). Δ65-1811 did not grow on KMB plates with a Cu^2+^ concentration of 5.6 mM, and Δ65-0387, Δ65-0388, and Δ65-0389 did not grow on KMB plates with a Cu^2+^ concentration of 6 mM. In group I strain pslb65, deletion of the *tolC* (*Aave_1811*) gene had a greater impact on copper tolerance compared to *cusC*-like (*Aave_0387*), *cusA*-like (*Aave_0388*), and *cusB*-like (*Aave_0389*). Conversely, Δ65-0038, Δ65-0039, Δ65-2798, and Δ65-4663 exhibited similar copper-tolerance levels to pslb65 (Figure 3). The copper MIC value for pslbtw14 was 2.8 mM (Figure 3a). Δ14-0034 did not grow on a KMB plate containing 0.3 mM copper, and Δ14-0033 lost its ability to grow at a copper concentration of 1.3 mM. At a copper concentration of 2.4 mM, Δ14-0032, Δ14-0387, Δ14-0388, Δ14-0389, and Δ14-1810 lost their ability to grow. However, the deletion of *Aave_0038*, *Aave_0039*, *Aave_1811*, *Aave_2798,* and *Aave_4663* genes in pslbtw14 did not affect the copper-tolerance level of the strains. Overall, strain pslb65 exhibited greater tolerance to copper stress compared to pslbtw14. The deletion mutants of the same gene in strain pslb65 generally showed lower copper sensitivity compared to pslbtw14, except for *Aave_0033*. The copper MIC values for Δ65-0033 and Δ14-0033 were both 1.3 mM. Furthermore, the deletion of the *Aave_0034* gene resulted in the loss of basic copper tolerance in both pslb65 and pslbtw14. The gene organization of the copper-tolerant genes characterized in this study is shown in Figure 4.

### 3.4. Copper-Tolerant Phenotype of the Complementary Strains

The *copA* (*Aave_0034*), *copZ* (*Aave_0033*), *cueR* (*Aave_0032*), and *cueO* (*Aave_1810*) genes showed 100% sequence identity between pslb65 and pslbtw14. However, the *cusA*-like (*Aave_0388*) gene had seven single nucleotide polymorphisms (SNPs), the *cusB*-like (*Aave_0389*) gene had three SNPs, the *cusC*-like (*Aave_0387*) gene had six SNPs, and the *tolC* (*Aave_1811*) gene had two SNPs. Subsequent analysis of the amino acid sequences revealed that these SNPs in the *cusA*-like, *cusB*-like, *cusC*-like, and *tolC* homologous genes resulted in differences in the amino acid sequences between *A. citrulli* strain pslb65 and pslbtw14. To investigate whether these genetic differences contributed to the distinct copper sensitivities of the two groups, this study used pslb65 as a template to construct complementary strains of mutant strains, and measured copper MIC of these strains.

**Figure 5:**
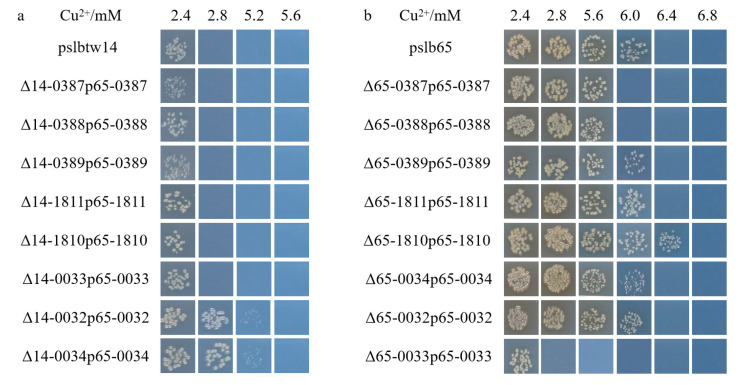
Colony growth of tested strains on KMB solid medium with varying concentrations of copper after 6 days of incubation. (**a**) Colony growth of representative *Acidovorax citrulli* group II strain pslbtw14 and its complementary strains. (**b**) Colony growth of representative *A*. *citrulli* group I strain pslb65 and its complementary strains. The notation p65-0032 represents pBBR1MCS-2 carrying the *Aave_0032* gene of pslb65, the complementary strain Δ14-0032p65-0032 represents the mutant strain Δ14-0032 carrying p65-0032, and the complementary strain Δ65-0032p65-0032 represents the mutant strain Δ65-0032 carrying p65-0032. Similar notations apply to the other strains. The strains were cultured in KMB liquid medium at 28 °C and 220 r/min for 12 h, and the bacterial suspensions were diluted to 3 × 10^4^ CFU/mL. A 10 µL bacterial suspension was spotted on KMB solid medium mixed with varying concentrations of CuSO_4_·5H_2_O. The picture shows the colony growth after 6 days of culture.

The copper-tolerance levels of the complementary strains Δ65-0032p65-0032 (Δ65-0032 complemented with *Aave_0032* gene of pslb65, similar below), Δ65-0034p65-0034, Δ65-1811p65-1811, and Δ65-0389p65-0389 were restored to the wildtype level (Figure 5b). However, Δ65-0387p65-0387 and Δ65-0388p65-0388 did not recover their copper sensitivity. The copper tolerance of Δ65-0033p65-0033 was partially restored but did not reach the wildtype level. Surprisingly, the copper-tolerance level of Δ65-1810p65-1810 was even higher than that of the wildtype strain. Comparing the complementary strains Δ14-0033p65-0033, Δ14-1810p65-1810, Δ14-1811p65-1811, Δ14-0387p65-0387, Δ14-0388p65-0388, and Δ14-0389p65-0389 with the wildtype strain pslbtw14, their copper-tolerance levels were consistent (Figure 5a). Δ14-0034p65-0034 and Δ14-0032p65-0032 exhibited higher copper-tolerance levels compared to pslbtw14. (Figure 5a).

### 3.5. Analysis of Copper-Tolerance-Related Genes Expression in Wildtype Strains

**Figure 6:**
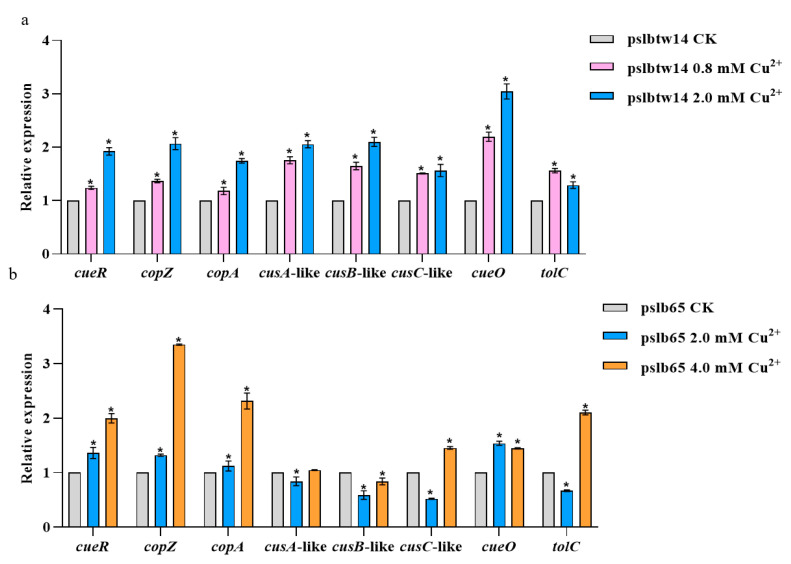
The expression levels of copper-tolerance-related genes in pslb65 and pslbtw14 when exposed to different copper concentrations. The control group (CK) received no copper treatment. The *rpoB* gene was used as an internal reference gene. Each treatment had three replicates, and the experiment was conducted three times. Error bars represent standard errors of the means, and asterisks denote significant differences (*p* < 0.05, two-way ANOVA test, Dunnett’s multiple comparisons test) (**a**,**b**).

The expression levels of copper-tolerance-related genes in pslb65 and pslbtw14 after copper treatment were measured, with a copper-free treatment as the control. Significant differences were observed in the expression levels of these genes under copper stress. At 0.8 and 2.0 mM Cu^2+^ concentrations, *cueR*, *copZ*, *copA*, *cusC*-like, *cusA*-like, *cusB*-like, *cueO,* and *tolC* were significantly upregulated in pslbtw14 (Figure 6a). However, in pslb65, *cusC*-like, *cusA*-like, *cusB*-like, and *tolC* were significantly downregulated at a low copper concentration (2.0 mM), but at a high copper concentration (4.0 mM), all genes except *cusA*-like and *cusB*-like were significantly upregulated (Figure 6b).

At a concentration of 2.0 mM Cu^2+^, the expression levels of copper-tolerance-related genes in pslb65 and pslbtw14 differed, with the genes in pslbtw14 showing significant upregulation. Conversely, the expression levels of *cusC*-like, *cusA*-like, *cusB*-like, and *tolC* genes in pslb65 were significantly downregulated.

### 3.6. Results of Analysis on the Expression of Copper-Tolerance-Related Genes in Mutant Strains

**Figure 7:**
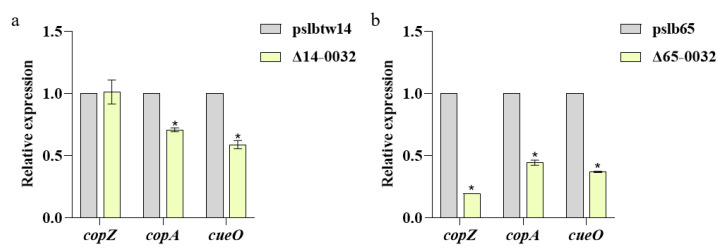
The expression levels of copper-tolerance-related genes of Δ14-0032 and Δ65-0032 following treatment with 1.6 mM copper. The *rpoB* gene was used as an internal reference gene. Each treatment had three replicates, and the experiment was conducted three times. Error bars represent standard errors of the means, and asterisks denote significant differences (*p* < 0.05, two-way ANOVA test, Dunnett’s multiple comparisons test). (**a**) The expression levels of copper-tolerance-related genes in pslbtw14 and Δ14-0032 when exposed to 1.6 mM copper. (**b**) The expression levels of copper-tolerance-related genes in pslb65 and Δ65-0032 when exposed to 1.6 mM copper.

The copper-tolerance-related genes of strains pslb65, Δ65-0032, pslbtw14, and Δ14-0032 were analyzed using qRT-PCR after treatment with 1.6 mM copper-containing KMB liquid medium. Compared to pslbtw14, the expression levels of *copA* and *cueO* in Δ14-0032 were significantly downregulated, while the expression levels of *copZ* remained unchanged (Figure 7a). However, in the group I strain, the expression levels of *copA*, *cueO* and *copZ* in Δ65-0032 were significantly downregulated compared with pslb65 (Figure 7b), with a much greater decrease compared to Δ14-0032.

## 4. Discussion

In this study, we investigated the copper tolerance of different strains of *A. citrulli* and identified various copper-tolerance genes. The results showed that group I strains exhibited higher copper tolerance compared to group II strains, as evidenced by their higher copper MIC values. This observation was further supported by the growth curves, which showed that group I strains had better growth compared to group II strains. Based on these results, pslb65 from group I and pslbtw14 from group II were selected as representative strains for subsequent experiments.

To further understand the role of putative copper-tolerance genes, 24 deletion mutants were constructed in pslb65 and pslbtw14. Our results showed that the deletion of certain genes (detailed below) greatly decreased copper tolerance. In contrast, the deletion of *cusA* (*Aave_0038*), *zneB* (*Aave_0039*), *cusB* (*Aave_4663*), and *gntR* (*Aave_2798*) genes in pslb65 and pslbtw14 did not impact their copper-tolerance levels. Overall, pslb65 exhibited greater tolerance to copper stress compared to pslbtw14, and the deletion mutants of the same gene in pslb65 generally showed lower copper sensitivity compared to pslbtw14.

The deletion of the *copA* (*Aave_0034*) and *copZ* (*Aave_0033*) genes resulted in a substantial reduction in copper tolerance in both group I and II strains of *A. citrulli*. Deleting *cueR* (*Aave_0032*) or *cueO* (*Aave_1810*) genes in pslbtw14 had a minor impact on copper tolerance, while deleting these genes in pslb65 reduced copper resistance by more than half. Therefore, we conclude that *copA* (*Aave_0034*), *copZ* (*Aave_0033*), *cueR* (*Aave_0032*), and *cueO* (*Aave_1810*) genes are major contributors to copper tolerance in *A. citrulli*. In particular, the deletion of the *copA* (*Aave_0034*) gene in the two representative strains led to almost complete loss of copper tolerance in the mutant strains, highlighting its core role in copper tolerance of *A. citrulli*. This finding aligns with the role of CopA in *E. coli*, where it functions as a central component of copper homeostasis under aerobic and anaerobic conditions and is responsible for cytoplasmic copper homeostasis [13]. Thus, we speculate that the development of *copA* inhibitors could offer improved control of BFB.

The *cusC*-like (*Aave_0387*), *cusA*-like (*Aave_0388*), and *cusB*-like (*Aave_0389*) genes had SNPs between group I strain pslb65 and group II strain pslbtw14. Deletion of these genes slightly decreased copper tolerance in strains pslb65 and pslbtw14. This suggests that these genes have a minor impact on copper tolerance of *A. citrulli*.

The *tolC* (*Aave_1811*) gene had 2 SNPs between group I strain pslb65 and group II strain pslbtw14, and their proteins have one amino acid difference. Interestingly, deleting *tolC* (*Aave_1811*) in pslbtw14 did not affect copper tolerance, whereas its deletion in pslb65 noticeably reduced copper tolerance from 6.4 mM to 5.6 mM. This suggests that this gene may only be active in pslb65, and that group I strain pslb65 possesses more copper-tolerance mechanisms than group II strain pslbtw14. In *E. coli*, the TolC protein is also involved in other efflux systems such as EmrAB-TolC or MacAB-TolC of the major facilitator superfamily (MFS) and ATP-binding cassette (ABC) superfamilies, respectively [31,32]. Therefore, it is possible that genes related to TolC efflux pumps may play a role in copper tolerance in *A. citrulli*. After treatment with 4.0 mM copper, the expression of *tolC* gene in pslb65 was significantly upregulated, suggesting a crucial role in group I strain pslb65. This effect was more pronounced compared to *cusC*-like, *cusA*-like, and *cusB*-like genes. This indicates that pslb65 may have a more efficient mechanism for exporting periplasmic toxic ions to the extracellular system, potentially involving other efflux pumps that work in conjunction with TolC to form efflux pumps.

The qRT-PCR results showed that at a copper concentration of 2.0 mM, the expression levels of copper-tolerance-related genes in pslbtw14 were significantly upregulated, while the expression levels of *cusC*-like, *cusA*-like, *cusB*-like, and *tolC* in pslb65 were significantly downregulated. The results indicated distinct differences in the expression of copper-related genes in pslb65 and pslbtw14 under copper stress. In addition, the expression levels of copper-tolerance-related genes in pslbtw14 treated with different copper concentrations indicated that at low and high copper concentrations, pslbtw14 seemed to rely on CueO to reduce the copper toxicity. However, the expression levels of copper-tolerance-related genes in pslb65 under different copper concentrations indicated that, at low copper concentrations, pslb65 relied on CueO to reduce the toxicity of copper, while at high copper concentrations, it mainly depended on the export of copper ions for detoxification. Overall, the varied expression of copper-tolerance-related genes in pslb65 and pslbtw14 suggests different strategies for coping with copper stress, potentially contributing to their differing copper sensitivities.

The deletion of copper efflux regulator *cueR* gene significantly reduced the expression levels of *copA*, *cueO,* and *copZ* genes in Δ65-0032 under copper stress, while the expression levels of *copZ* genes in Δ14-0032 remained relatively stable. The downregulation of copper-tolerance-related genes was more pronounced in Δ65-0032 compared to Δ14-0032, indicating a potential divergence in the role of the *cueR* gene between these two strains.

Some complementary strains restored copper tolerance to the wildtype level, while others showed increased or decreased tolerance compared to the wildtype. Previous research on *E. coli* has shown that the copper chaperone protein CopZ and the copper efflux protein CopA function together to remove excess copper ions from the cytoplasm [33]. In *A. citrulli*, we hypothesized that CopZ binds to copper ions and transfers them to CopA for export. In the complementary strain Δ65-0033p65-0033, overexpression of the *copZ* (*Aave_0033*) gene resulted in an accumulation of copper chaperone CopZ carrying copper ions. However, the removal rate of copper ions from the cytoplasm was limited by CopA, resulting in a slower elimination of copper ions. This may explain why this complementary strain only partially restored its copper-tolerance level. On the other hand, the copper-tolerance level of the complementary strain Δ65-0033p65-0033 restored to the level of wildtype strain pslbtw14, suggesting that overexpression of the *copZ* gene can only enable the strain to achieve a maximum copper-tolerance level of 2.8 mM.

Increasing the copy number of the *copA* (*Aave_0034*) gene has been shown to enhance the copper tolerance of strains in *Acidithiobacillus thiooxidans* [34]. In this study, the copper tolerance of Δ14-0034p65-0034 was approximately double that of pslbtw14, likely due to the overexpression of the *copA* gene carried by the complementary plasmid p65-0034. However, the copper-tolerance level of Δ65-0034p65-0034 was similar to that of pslb65, suggesting that overexpression of the *copA* gene can only increase copper tolerance up to a maximum level of 6.4 mM.

Previous studies have suggested that CueR can interact with P-type ATPase (CopA) and multicopper oxidase (CueO) in *A. citrulli* [35]. The presence of a typical palindrome motif in the promoter region of *copA*, which binds to CueR, suggests that CueR may positively regulate *copA*, resulting in an increase in *copA* gene copies [35]. This increase enhances the active pumping of intracellular copper ions and improves copper tolerance. The higher copper-tolerance level observed in the complementary strain Δ14-0032p65-0032 compared to pslbtw14 may be due to the overexpression of the *cueR* (*Aave_0032*) gene. The copper-tolerance level of Δ65-0032p65-0032 was consistent with that of pslb65, suggesting that overexpression of the *cueR* (*Aave_0032*) gene can only achieve a maximum copper-tolerance level of 6.4 mM.

In addition, the complementary strain Δ65-1810p65-1810 exhibited enhanced copper tolerance compared to pslb65, which can be attributed to the overexpression of the *cueO* (*Aave_1810*) gene. CueO oxidizes highly toxic Cu^+^ ions in the cytoplasm to less toxic Cu^2+^ ions, reducing overall copper toxicity. Additionally, CueO also oxidizes certain chelating peptides containing reduced metal ions in the periplasmic space, further reducing the rate of Cu^2+^ reduction [13]. Consequently, the copper-tolerance level of Δ65-1810p65-1810 surpassed that of the wildtype strain. However, the copper-tolerance level of Δ14-1810p65-1810 remained similar to pslbtw14, suggesting that the impact of *cueO* (*Aave_1810*) on pslb65 is greater than on pslbtw14.

Overall, our study provides valuable insights into the copper tolerance of different strains of *A. citrulli* and identifies key genes associated with copper tolerance. These findings suggest that the differential copper sensitivity between the two groups of *A. citrulli* strains may be influenced by several factors, including additional mechanisms for copper ion removal, variations in the expression levels of copper-tolerance-related genes, and the complex genomic basis for copper tolerance. Further studies are needed to fully understand the mechanisms underlying copper tolerance in *A. citrulli* and its implications for pathogenicity.

## Figures and Tables

**Table 1:** Primers used for quantitative real-time PCR.

Primers	Primer Sequence (5′-3′)	Length/bp	Source
*rpoB*-F	GCGACAGCGTGCTCAAAGTG	134	[30]
*rpoB*-R	GCCTTCGTTGGTGCGTTTCT		
*cueR*-QF	CGCATGGTCCGCCACTAC	173	[19]
*cueR*-QR	TCCTGCCAGAGCCCGAG		
*copZ*-QF	TGACCTGCGGCCATTGC	118	[19]
*copZ*-QR	CGAGGGCTGTCGCTTTCC		
*copA*-QR	TGTCGCTGTGGCTGTGGTTC	144	[19]
*copA*-QF	CTTCCGTGGTCTGCCGCTTG		
*cusA*-QR	AGGGCTTCAACCTGTCGCT	143	This study
*cusA*-QF	GTTGAGTTGCCCCTTGACG		
*cusB*-QR	TTCACGGAAGGCAGCGAC	200	This study
*cusB*-QF	ACCGCGTTGTCGTACTCCTG		
*cusC*-QR	GCTGCCAACGCCAACATC	111	This study
*cusC*-QF	GCCGCCCTTGAACAGACC		
*cueO*-QR	GACCAACCACCCCATCCAC	187	This study
*cueO*-QF	GTGGTGGCTCTTGTGGCAGT		
*tolC*-QR	GGCCATTCCGAAATCAAGC	101	This study
*tolC*-QF	TCATTCACAAGCCCCTACGC		

## Data Availability

Data are contained within the article.

## Supplementary Materials

The following supporting information can be downloaded at https://www.mdpi.com/article/10.3390/microorganisms12040682/s1. Table S1: Strains and plasmids used in this study; Table S2: Primers used for construction of mutant and complementary strains. Figure S1: Validation of *Acidovorax citrulli* mutant strains using target-gene-specific primers; Figure S2: Validation of the species of mutant strains using *Acidovorax citrulli-specific* primers WFB1/WFB2; Figure S3: Validation of *Acidovorax citrulli-*complementary strains using target-gene-specific primers; Figure S4: Validation of *Acidovorax citrulli*-complementary strains using Kan-F/Kan-R primers.
