# Supplementary material for: Comparison of Copper-Tolerance Genes between Different Groups of Acidovorax citrulli
Source: Microorganisms. 2024 Mar 28;12(4):682. doi: 10.3390/microorganisms12040682 (PMC11052375; doi:10.3390/microorganisms12040682)
Supplement: Supplementary file 1 [file microorganisms-12-00682-s001.zip › microorganisms-2869949-supplementary.pdf]

Supplementary Materials

Supplementary Materials: The following supporting information can be downloaded at: www.mdpi.com/xxx/s1, Table S1: Strains and plasmids used in this study; Table S2: Primers used for construction of mutant and complementary strains. Figure S1: Validation of *Acidovorax citrulli* mutant strains using target-gene-specific primers; Figure S2: Validation of the species of mutant strains using *Acidovorax citrulli*-specific primers WFB1/WFB2; Figure S3: Validation of *Acidovorax citrulli-complementary* strains using target-gene-specific primers; Figure S4: Validation of *Acidovorax citrulli-complementary* strains using Kan-F/Kan-R primers.

Table S1. Strains and plasmids used in this study.

Strains or Plasmids	Description	Source
Strains		
A. citrulli pslb9	Amp^r, the wildtype strain (group I)	Lab preservation
A. citrulli pslb65	Amp^r, the wildtype strain (group I)	Lab preservation
A. citrulli pslbtw14	Amp^r, the wildtype strain (group II)	Lab preservation
A. citrulli Aac5	Amp^r, the wildtype strain (group II)	Lab preservation
A. citrulli pslbtw32	Amp^r, the wildtype strain (group II)	Lab preservation
A. citrulli pslbtw38	Amp^r, the wildtype strain (group II)	Lab preservation
Δ14-0032	Amp^r, *Aave_0032* gene deletion mutant derived from pslbtw14	This study
Δ65-0032	Amp^r, *Aave_0032* gene deletion mutant derived from pslb65	This study
Δ14-0033	Amp^r, *Aave_0033* gene deletion mutant derived from pslbtw14	This study
Δ65-0033	Amp^r, *Aave_0033* gene deletion mutant derived from pslb65	This study
Δ14-0034	Amp^r, *Aave_0034* gene deletion mutant derived from pslbtw14	This study
Δ65-0034	Amp^r, *Aave_0034* gene deletion mutant derived from pslb65	This study
Δ14-0038	Amp^r, *Aave_0038* gene deletion mutant derived from pslbtw14	This study
Δ65-0038	Amp^r, *Aave_0038* gene deletion mutant derived from pslb65	This study
Δ14-0039	Amp^r, *Aave_0039* gene deletion mutant derived from pslbtw14	This study
Δ65-0039	Amp^r, *Aave_0039* gene deletion mutant derived from pslb65	This study
Δ14-0387	Amp^r, *Aave_0387* gene deletion mutant derived from pslbtw14	This study
Δ65-0387	Amp^r, *Aave_0387* gene deletion mutant derived from pslb65	This study
Δ14-0388	Amp^r, *Aave_0388* gene deletion mutant derived from pslbtw14	This study
Δ65-0388	Amp^r, *Aave_0388* gene deletion mutant derived from pslb65	This study
Δ14-0389	Amp^r, *Aave_0389* gene deletion mutant derived from pslbtw14	This study
Δ65-0389	Amp^r, *Aave_0389* gene deletion mutant derived from pslb65	This study

Δ14-1810	Amp^r, *Aave_1810* gene deletion mutant derived from pslbtw14	This study
Δ65-1810	Amp^r, *Aave_1810* gene deletion mutant derived from pslb65	This study
Δ14-1811	Amp^r, *Aave_1811* gene deletion mutant derived from pslbtw14	This study
Δ65-1811	Amp^r, *Aave_1811* gene deletion mutant derived from pslb65	This study
Δ14-2798	Amp^r, *Aave_2798* gene deletion mutant derived from pslbtw14	This study
Δ65-2798	Amp^r, *Aave_2798* gene deletion mutant derived from pslb65	This study
Δ14-4663	Amp^r, *Aave_4663* gene deletion mutant derived from pslbtw14	This study
Δ65-4663	Amp^r, *Aave_4663* gene deletion mutant derived from pslb65	This study
Δ65-0032p65-0032	Amp^r, Kan^r, Δ65-0032 complemented with *Aave_0032* gene of pslb65	This study
Δ14-0032p65-0032	Amp^r, Kan^r, Δ14-0032 complemented with *Aave_0032* gene of pslb65	This study
Δ65-0033p65-0033	Amp^r, Kan^r, Δ65-0033 complemented with *Aave_0033* gene of pslb65	This study
Δ14-0033p65-0033	Amp^r, Kan^r, Δ14-0033 complemented with *Aave_0033* gene of pslb65	This study
Δ65-0034p65-0034	Amp^r, Kan^r, Δ65-0034 complemented with *Aave_0034* gene of pslb65	This study
Δ14-0034p65-0034	Amp^r, Kan^r, Δ14-0034 complemented with *Aave_0034* gene of pslb65	This study
Δ65-0387p65-0387	Amp^r, Kan^r, Δ65-0387 complemented with *Aave_0387* gene of pslb65	This study
Δ14-0387p65-0387	Amp^r, Kan^r, Δ14-0387 complemented with *Aave_0387* gene of pslb65	This study
Δ65-0388p65-0388	Amp^r, Kan^r, Δ65-0388 complemented with *Aave_0388* gene of pslb65	This study
Δ14-0388p65-0388	Amp^r, Kan^r, Δ14-0388 complemented with *Aave_0388* gene of pslb65	This study
Δ65-0389p65-0389	Amp^r, Kan^r, Δ65-0389 complemented with *Aave_0389* gene of pslb65	This study
Δ14-0389p65-0389	Amp^r, Kan^r, Δ14-0389 complemented with *Aave_0389* gene of pslb65	This study
Δ65-1810p65-1810	Amp^r, Kan^r, Δ65-1810 complemented with *Aave_1810* gene of pslb65	This study
Δ14-1810p65-1810	Amp^r, Kan^r, Δ14-1810 complemented with *Aave_1810* gene of pslb65	This study
Δ65-1811p65-1811	Amp^r, Kan^r, Δ65-1811 complemented with *Aave_1811* gene of pslb65	This study
Δ14-1811p65-1811	Amp^r, Kan^r, Δ14-1811 complemented with *Aave_1811* gene of pslb65	This study
E. coli DH5*α*	Φ80*lac*ZΔM15Δ (*lac*ZYA-argF) U169*end*A1*rec*A1 *hsd*R17*sup*E44 thi-1 *gyr*A96 *rel*A1 *pho*A	Tiangen, China
plasmids		

pK18mobsacB	Kan^r, containing the *sacB* sucrose lethal site	Lab collection
pBBR1MCS-2	Kan^r, broad host range expression vector with *lac* promoter	Lab collection
pK18-0032(14)	Kan^r, suicide-recombinant vector used to generate the mutant strain Δ14-0032	This study
pK18-0032(65)	Kan^r, suicide-recombinant vector used to generate the mutant strain Δ65-0032	This study
pK18-0033(14)	Kan^r, suicide-recombinant vector used to generate the mutant strain Δ14-0033	This study
pK18-0033(65)	Kan^r, suicide-recombinant vector used to generate the mutant strain Δ65-0033	This study
pK18-0034(14)	Kan^r, suicide-recombinant vector used to generate the mutant strain Δ14-0034	This study
pK18-0034(65)	Kan^r, suicide-recombinant vector used to generate the mutant strain Δ65-0034	This study
pK18-0038(14)	Kan^r, suicide-recombinant vector used to generate the mutant strain Δ14-0038	This study
pK18-0038(65)	Kan^r, suicide-recombinant vector used to generate the mutant strain Δ65-0038	This study
pK18-0039(14)	Kan^r, suicide-recombinant vector used to generate the mutant strain Δ14-0039	This study
pK18-0039(65)	Kan^r, suicide-recombinant vector used to generate the mutant strain Δ65-0039	This study
pK18-0387(14)	Kan^r, suicide-recombinant vector used to generate the mutant strain Δ14-0387	This study
pK18-0387(65)	Kan^r, suicide-recombinant vector used to generate the mutant strain Δ65-0387	This study
pK18-0388(14)	Kan^r, suicide-recombinant vector used to generate the mutant strain Δ14-0388	This study
pK18-0388(65)	Kan^r, suicide-recombinant vector used to generate the mutant strain Δ65-0388	This study
pK18-0389(14)	Kan^r, suicide-recombinant vector used to generate the mutant strain Δ14-0389	This study
pK18-0389(65)	Kan^r, suicide-recombinant vector used to generate the mutant strain Δ65-0389	This study
pK18-1810(14)	Kan^r, suicide-recombinant vector used to generate the mutant strain Δ14-1810	This study
pK18-1810(65)	Kan^r, suicide-recombinant vector used to generate the mutant strain Δ65-1810	This study
pK18-1811(14)	Kan^r, suicide-recombinant vector used to generate the mutant strain Δ14-1811	This study
pK18-1811(65)	Kan^r, suicide-recombinant vector used to generate the mutant strain Δ65-1811	This study
pK18-2798(14)	Kan^r, suicide-recombinant vector used to generate the mutant strain Δ14-2798	This study
pK18-2798(65)	Kan^r, suicide-recombinant vector used to generate the mutant strain Δ65-2798	This study
pK18-4663(14)	Kan^r, suicide-recombinant vector used to generate the mutant strain Δ14-4663	This study
pK18-4663(65)	Kan^r, suicide-recombinant vector used to generate the mutant strain Δ65-4663	This study

p65-0032	Kan^r, pBBR1MCS-2 carrying *Aave_0032* gene of pslb65	This study
p65-0033	Kan^r, pBBR1MCS-2 carrying *Aave_0033* gene of pslb65	This study
p65-0034	Kan^r, pBBR1MCS-2 carrying *Aave_0034* gene of pslb65	This study
p65-0387	Kan^r, pBBR1MCS-2 carrying *Aave_0387*gene of pslb65	This study
p65-0388	Kan^r, pBBR1MCS-2 carrying *Aave_0388* gene of pslb65	This study
p65-0389	Kan^r, pBBR1MCS-2 carrying *Aave_0389* gene of pslb65	This study
p65-1810	Kan^r, pBBR1MCS-2 carrying *Aave_1810* gene of pslb65	This study
p65-1811	Kan^r, pBBR1MCS-2 carrying *Aave_1811* gene of pslb65	This study

Note: Kan^r, Cm^r, and Amp^r indicate resistance to kanamycin, chloramphenicol, and ampicillin, respectively.

Table S2. Primers used for construction of mutant and complementary strains.

Primers	Primer Sequence (5′-3′)	Length/bp
0032-1F	CTATGACATGATTACGAATTCGGCTCGTAACCCGCATTG	587
0032-1R	CGTCAGGTATATCGCATTGCGACGGTGTAGCCCTCTTCG	
0032-2F	CGAAGAGGGCTACACCGTCGCAATGCGATATACCTGACG	530
0032-2R	ACGACGGCCAGTGCCAAGCTTATGCCCAGCATGTAGTCGA	
0032-F	TGGTCCGCCACTACGAGG	336
0032-R	CCGCCAGATCATCCAGGA	
0033-1F	CTATGACATGATTACGAATTCAGCACCAGCGGAAGCGACA	472
0033-1R	CCTCGTAGTGGCGGACCATGGCGGGATGCAGGGATGAT	
0033-2F	ATCATCCCTGCATCCCGCCATGGTCCGCCACTACGAGG	502
0033-2R	ACGACGGCCAGTGCCAAGCTTCGATGGAGGCGATGAGGAT	
0033-F	TGACCTGCGGCCATTGC	166
0033-R	GCAGCGACGGTGTAGCCCT	
0034-1F	CTATGACATGATTACGAATTCGTGTGGACATCCCTCTCGG	495
0034-1R	TGATGGGGCAAGCGTAACGCTTGACTCTGCCACCATGT	
0034-2F	ACATGGTGGCAGAGTCAAGCGTTACGCTTGCCCCATCA	434
0034-2R	ACGACGGCCAGTGCCAAGCTTGTTGTCCCTGTCCTGCCC	
0034-F	TCGCTGTGGCTGTGGTTC	518
0034-R	GCCGAAACCCTGTCCACT	
0038-1F	CTATGACATGATTACGAATTCGACCCGACCTCCCTCTGG	493
0038-1R	GCCTACCGACCACTGACCGGGGCCTCTTTCTTCGCGGT	
0038-2F	ACCGCGAAGAAAGAGGCCCCGGTCAGTGGTCGGTAGGC	542
0038-2R	ACGACGGCCAGTGCCAAGCTTTGCCATTCCAGGGTCTCCG	
0038-F	TGTTCTCCCACACGCAGTTC	360
0038-R	GGTTCTGGAAGCTCACCTTG	
0039-1F	CTATGACATGATTACGAATTCGCAGTGGGGCTACCAGTACC	406
0039-1R	GCCTCTTTCTTCGCGGTGGGAAGGCAGTGCTGTGGC	
0039-2F	GCCACAGCACTGCCTTCCCACCGCGAAGAAAGAGGC	450
0039-2R	ACGACGGCCAGTGCCAAGCTTGCCCTTTAGGCGGTAGCG	
0039-F	TCGAGCGCAACCTCAACC	534
0039-R	CGGCCATGGACTGGAACT	

0387-1F	CTATGACATGATTACGAATTCCGTGGAGTTCGCCAAGGA	473
0387-1R	GCCTGGAAAAGAGCGGAAGCGTTCGTAGGTGGGGAT	
0387-2F	ATCCCCACCTACGAACGCTTCCGCTCTTTTCCAGGC	432
0387-2R	ACGACGGCCAGTGCCAAGCTTAAAGGTAGATGGACCCCCG	
0387-F	GTCGGGTCTGTTCAAGGG	404
0387-R	CGCCGAGCACTTTGTAGA	
0388-1F	CTATGACATGATTACGAATTCAAGTGCCCAATCCCGAAC	487
0388-1R	TGAAGAAGGAACGCACCAGCGTGATGACCCATGCGAAGA	
0388-2F	TCTTCGCATGGGTCATCACGCTGGTGCGTTCCTTCTTCA	554
0388-2R	ACGACGGCCAGTGCCAAGCTTTCCCAGGCAGAGACCGC	
0388-F	CTCACGACGGAAAACCCC	299
0388-R	TTGAGTTGCCCCTTGACG	
0389-1F	CTATGACATGATTACGAATTCCGCACGGATTCGTCTTTCT	494
0389-1R	CTGGGGGTTACTGCTTCGCAGGGGAAAAGGCTGTCGC	
0389-2F	GCGACAGCCTTTTCCCCTGCGAAGCAGTAACCCCCAG	460
0389-2R	ACGACGGCCAGTGCCAAGCTTGCATGGCGAACATCAGGAA	
0389-F	ACAAGCAGGCTGCAAAGG	349
0389-R	TCCAGCACGACCCAGTTG	
1810-1F	CTATGACATGATTACGAATTCTGCGTAGGGGCTTGTGAA	430
1810-1R	GATGAAGGTGTGACGCGCTTGTCTGCGTTGCATGGGT	
1810-2F	ACCCATGCAACGCAGACAAGCGCGTCACACCTTCATC	431
1810-2R	ACGACGGCCAGTGCCAAGCTTAGGAGCCGAGCTTCTGGG	
1810-F	CGTCAACCTCTGGGGCTAC	245
1810-R	GCGGGTGGTACATGAAGG	
1811-1F	CTATGACATGATTACGAATTCCCCGTCTCCATCGTTCGTT	416
1811-1R	GGAGTGGGGGAGGACAGGCTGCGGTCATGGCTGGTG	
1811-2F	CACCAGCCATGACCGCAGCCTGTCCTCCCCCACTCC	426
1811-2R	ACGACGGCCAGTGCCAAGCTTAGGCGGTTGGTGACGAAG	
1811-F	GTGCGTAGGGGCTTGTGA	193
1811-R	GCGAGCATGCCGTTGTAG	
2798-1F	CTATGACATGATTACGAATTCTTCGTCGTAGTCGAGTTCCA	446
2798-1R	GAAATCCTGCCCATCCCCGGCGATTGACCTCATTCATT	
2798-2F	AATGAATGAGGTCAATCGCCGGGGATGGGCAGGATTTC	526
2798-2R	ACGACGGCCAGTGCCAAGCTTGCCATCAGGGCATCCAAAG	
2798-F	ATGGAGACCAGCGAAGCG	314
2798-R	GCGATGTCGTCGTTGCAG	
4663-1F	CTATGACATGATTACGAATTCAACACCTGGTCGATCGGG	481
4663-1R	CGGGGGGACAGCTTCTTCGGAAGTTGCGGTCGGTGT	
4663-2F	ACACCGACCGCAACTTCCGAAGAAGCTGTCCCCCCG	494
4663-2R	ACGACGGCCAGTGCCAAGCTTGATGGCGAAGGCGAGGAT	
4663-F	CGTCTATGTCGATCTGCCCG	280
4663-R	GTCGCCGTCGTTGAGGAA	
WFB1	GACCAGCCACACTGGGAC	360
WFB2	CTGCCGCACTCCAGCGA	
M13F	CAGGAAACAGCTATGAC	-
M13R	GTAAAACGACGGCCAGT	
Kan-F	CAAGATGGATTGCACGCA	738
Kan-R	CTTGAGCCTGGCGAACA	
H0032F	GTCGACGGTATCGATAAGCTTCCGAAGAGGGCTACACCGT	664
H0032R	CGCTCTAGAACTAGTGGATCCCGATGGAGGCGATGAGGA	
H0033F	GTCGACGGTATCGATAAGCTTTCCAGGGACTGCTCAGGC	543

H0033R	CGCTCTAGAACTAGTGGATCCGTAGTGGCGGACCATGCG	
H0034F	GTCGACGGTATCGATAAGCTTGGCAGGCTCCTTTTCAAAT	2464
H0034R	CGCTCTAGAACTAGTGGATCCACTGATGGGGCAAGCGTAA	
H0387F	GTCGACGGTATCGATAAGCTTCACGGATGGGGAATAGGC	1585
H0387R	CGCTCTAGAACTAGTGGATCCAGACCCGGTGCACAGGAA	
H0388F	GTCGACGGTATCGATAAGCTTCGAAGCAGTAACCCCCAGAA	3326
H0388R	CGCTCTAGAACTAGTGGATCCTCGTAGGTGGGGATGAAGCT	
H0389F	GTCGACGGTATCGATAAGCTTGGTCCTGCGACAGCCTTT	1367
H0389R	CGCTCTAGAACTAGTGGATCCCTCCGTGTTCTGGGGGTT	
H1810F	GTCGACGGTATCGATAAGCTTCCTGTCCTCCCCCACTCC	1615
H1810R	CGCTCTAGAACTAGTGGATCCATGGCGGGTTTCCTTTCC	
H1811F	GTCGACGGTATCGATAAGCTTGAGGGCACCAGCCATGAC	1528
H1811R	CGCTCTAGAACTAGTGGATCCCCGAAGAAGTGTCTGCGTTG	

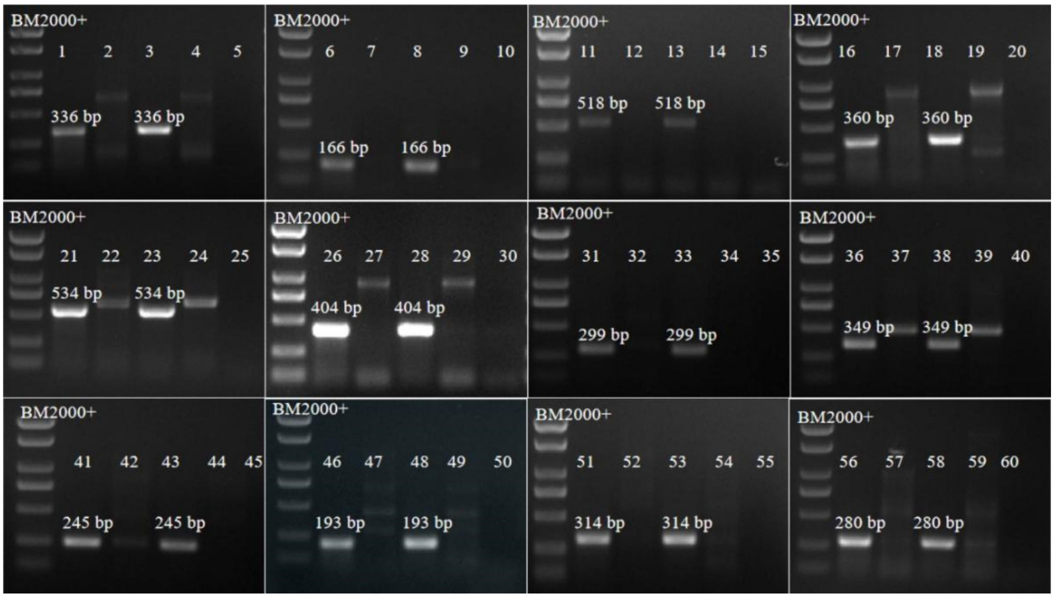

Figure S1. Validation of *Acidovorax citrulli* mutant strains using target-gene-specific primers. Lanes 1~5 correspond to the validation using primers 0032-F/0032-R with pslb65, Δ65-0032, pslbtw14, Δ14-0032, and the negative control as samples. Lanes 6~10 correspond to the validation using primers 0033-F/0033-R, with the same sample order as lanes 1~5. Lanes 11~15 correspond to the validation using primers 0034-F/0034-R. Lanes 16~20 correspond to the validation using primers 0038-F/0038-R. Lanes 21~25 correspond to the validation using primers 0039-F/0039-R. Lanes 26~30 correspond to the validation using primers 0387-F/0387-R. Lanes 31~35 correspond to the validation using primers 0388-F/0388-R. Lanes 36~40 correspond to the validation using primers 0389-F/0389-R. Lanes 41~45 correspond to the validation using primers 1810-F/1810-R. Lanes 46~50 correspond to the validation using primers 1811-F/1811-R. Lanes 51~55 correspond to the validation using primers 2798-F/2798-R. Lanes 56~60 correspond to the validation using primers 4663-F/4663-R.

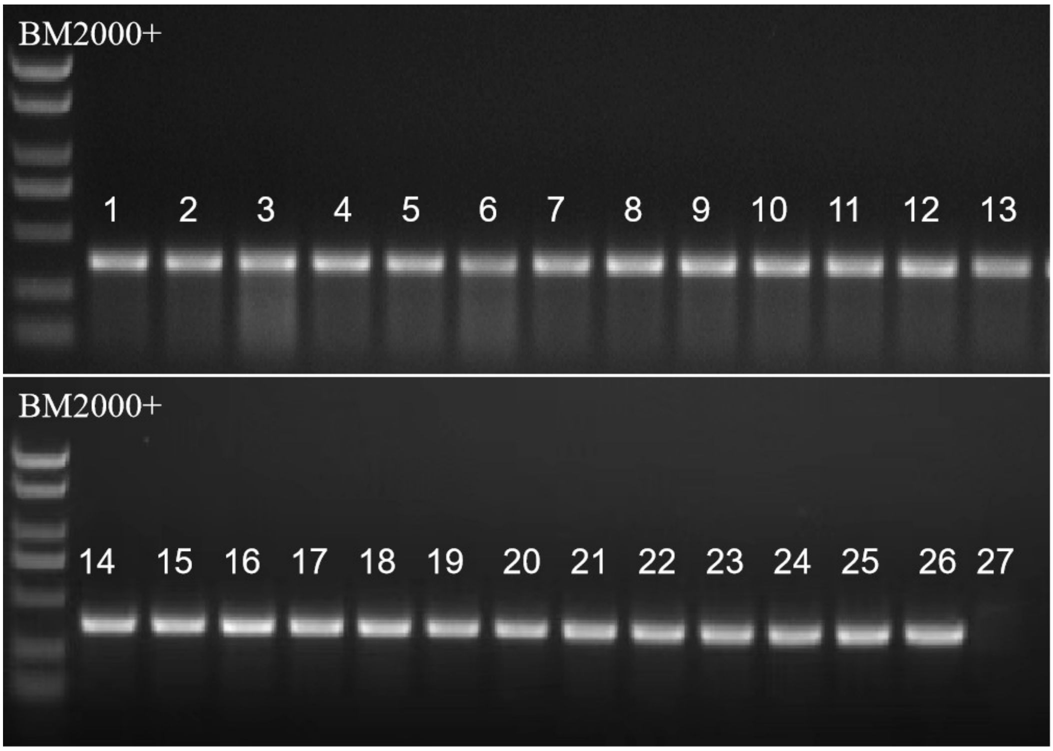

Figure S2. Validation of the species of mutant strains using *Acidovorax citrulli*-specific primers WFB1/WFB2. Lanes 1~27 correspond to the following strains: pslb65, Δ65-0032, Δ65-0033, Δ65-0034, Δ65-0038, Δ65-0039, Δ65-0387, Δ65-0388, Δ65-0389, Δ65-1810, Δ65-1811, Δ65-2798, Δ65-4663, pslbtw14, Δ14-0032, Δ14-0033, Δ14-0034, Δ14-0038, Δ14-0039, Δ14-0387, Δ14-0388, Δ14-0389, Δ14-1810, Δ14-1811, Δ14-2798, Δ14-4663, and the negative control.

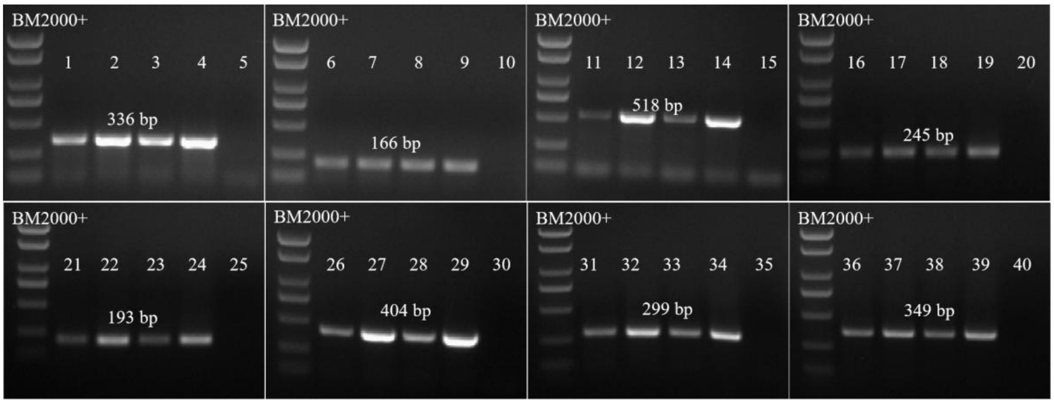

Figure S3. Validation of *Acidovorax citrulli-complementary* strains using target-gene-specific primers. Lanes 1~5 correspond to the validation using primers 0032-F/0032-R with pslb65, Δ65-0032p65-0032, pslbtw14, Δ14-0032p65-0032, and the negative control as samples. Lanes 6~10 correspond to the validation using primers 0033-F/0033-R, with the same sample order as lanes 1~5. Lanes 11~15 correspond to the validation using primers 0034-F/0034-R. Lanes 16~20 correspond to the validation using primers 1810-F/1810-R. Lanes 21~25 correspond to the validation using primers 1811-F/1811-R. Lanes 26~30 correspond to the validation using primers 0387-F/0387-R. Lanes 31~35 correspond to the validation using primers 0388-F/0388-R. Lanes 36~40 correspond to the validation using primers 0389-F/0389-R.

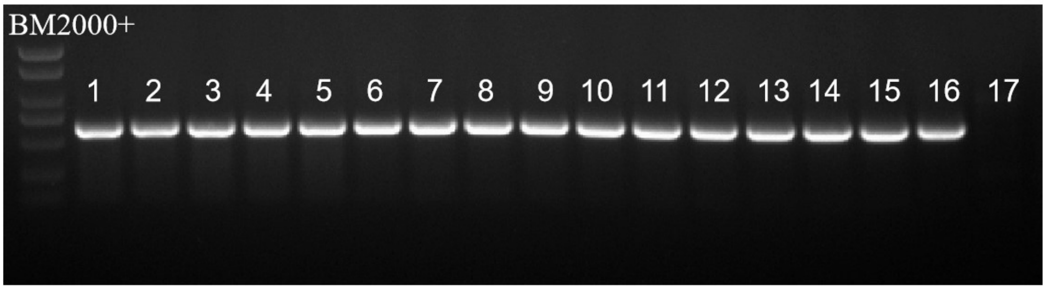

Figure S4. Validation of *Acidovorax citrulli-complementary* strains using Kan-F/Kan-R primers. Lanes 1~17 correspond to the following samples: Δ65-0032p65-0032, Δ14-0032p65-0032, Δ65-0033p65-0033, Δ14-0033p65-0033, Δ65-0034p65-0034, Δ14-0034p65-0034, Δ65-1810p65-1810, Δ14-0033p65-1810, Δ65-1811p65-1811, Δ14-1811p65-1811, Δ65-0387p65-0387, Δ14-0387p65-0387, Δ65-0388p65-0388, Δ14-0388p65-0388, Δ65-0389p65-0389, Δ14-0389p65-0389, and the negative control.
